# Incidence and associated risk factors of nontuberculous mycobacterial infection in patients with depression

**DOI:** 10.1371/journal.pone.0290271

**Published:** 2023-08-17

**Authors:** Woo Kyung Ryu, Jakyung Lee, Youngmok Park, Inkyung Jung, Young Ae Kang

**Affiliations:** 1 Division of Pulmonary and Critical Care Medicine, Department of Internal Medicine, Severance Hospital, Yonsei University College of Medicine, Seoul, Korea; 2 Department of Biostatistics and Computing, Yonsei University Graduate School, Seoul, Korea; 3 Division of Biostatistics, Department of Biomedical Systems Informatics, Yonsei University College of Medicine, Seoul, Korea; Kyung Hee University School of Medicine, REPUBLIC OF KOREA

## Abstract

**Background:**

It has been reported that the risk of mental health problems such as anxiety or depression increases in patients with nontuberculous mycobacterial (NTM) infection. However, no studies have investigated whether the incidence of NTM infection increases in patients with depression. This study aimed to investigate the incidence of NTM infection in patients with depression and evaluate the association between NTM infection and depression stratified by age and sex.

**Methods:**

Data from 2002 to 2013 were collected from patients aged ≥ 20 years in the National Health Insurance Service-National Sample Cohort database. Patients with and without depression aged over 20 years were matched with 1 to 4 by sex, age, and year of diagnosis. The incidence rate was calculated in 100,000 person-years, and a multivariable subdistribution hazard model was used to evaluate the adjusted hazard ratio (aHR) for the development of NTM infection.

**Results:**

We included 37,554 individuals (12,752 men and 24,802 women) and 149,213 controls in the depression and non-depression groups, respectively. The cumulative incidence of NTM infection did not differ significantly between the depression and non-depression groups during the follow-up period (22.2 vs. 24.5 per 100,000 person-years, p = 0.571). The age- and sex-stratified effects on the incidence of NTM infection were not significantly higher in patients with depression than in those without depression. After adjusting for covariates including age, sex, comorbidity, income, and region, the risk of NTM infection did not significantly differ between the depression and non-depression groups (aHR 0.83, 95% confidence interval 0.58–1.17).

**Conclusion:**

The incidence of NTM infections in patients with depression was not significantly higher than that in patients without depression. However, due to the small number of NTM infections, we might have underestimated the differences between the two groups. Further studies are needed to identify factors associated with NTM pulmonary disease in patients with depression.

## Introduction

Nontuberculous mycobacterial (NTM) species are opportunistic pathogens that are mainly found in soil and water systems and can cause progressive pulmonary disease [1]. The incidence and prevalence of NTM-pulmonary disease (PD) are increasing worldwide, especially in the elderly population [2, 3]. As epidemiological significance has been emphasized, clinical awareness and our understanding of NTM-PD have improved. However, the treatment success rate of NTM-PD is approximately 40–60% for *Mycobacterium avium* complex and 30–40% for *Mycobacterium abscessus* pulmonary disease, which usually shows a chronic intractable course [4, 5].

Most patients with chronic diseases have mental health problems, such as depression, owing to the long-term course of the disease. A higher incidence of depression has been reported in patients with chronic medical conditions such as hypertension, diabetes, and coronary heart disease [6]. Likewise, these psychiatric problems are more common in patients with chronic lung diseases, including chronic obstructive pulmonary disease, idiopathic pulmonary fibrosis, and pulmonary tuberculosis [7–9]. It has been also reported that the risk of anxiety and depression increases in patients with NTM-PD. Approximately 20% of the NTM-PD patients had anxiety or depression at the time of diagnosis [10, 11].

According to the World Health Organization, approximately 5% of the adults suffers from depression in the world [12]. Depression may also affect the incidence of other medical conditions and is bidirectionally associated with comorbidities. Previous studies have shown that depression increases the risk of several chronic illnesses, such as cardiovascular diseases, diabetes, and respiratory diseases, including tuberculosis [13, 14]. However, no studies have investigated whether the incidence of NTM-PD increases in patients with depression.

This study aimed to investigate the incidence of NTM-PD in patients with depression and evaluate the association between NTM-PD and depression stratified by age and sex.

## Materials and methods

### Data source

Data were obtained from National Health Insurance Service-National Sample Cohort (NHIS-NSC) 1.0 database. The NHIS-NSC is a population-based cohort, with a sample representing approximately 2.2% of the general Korean population of 46,605,433. The cohort data included medical service claims, pharmacy claims, and demographic information, such as age, sex, household income, insurance type, region of residence, and mortality information. A detailed description of these data was provided in a previous study [15].

### Study population

This population-based retrospective cohort study initially considered 737,226 individuals aged > 20 years, from January 2002 to December 2013. Participants were classified into depression (n = 42,969) and non-depression (n = 694,257) groups based on the diagnosis of depression. Patients diagnosed with depression in 2002 (washout period, n = 5,396) or who had a history of NTM infection before the diagnosis of depression (n = 19) were excluded from the depression group.

**Fig 1:**
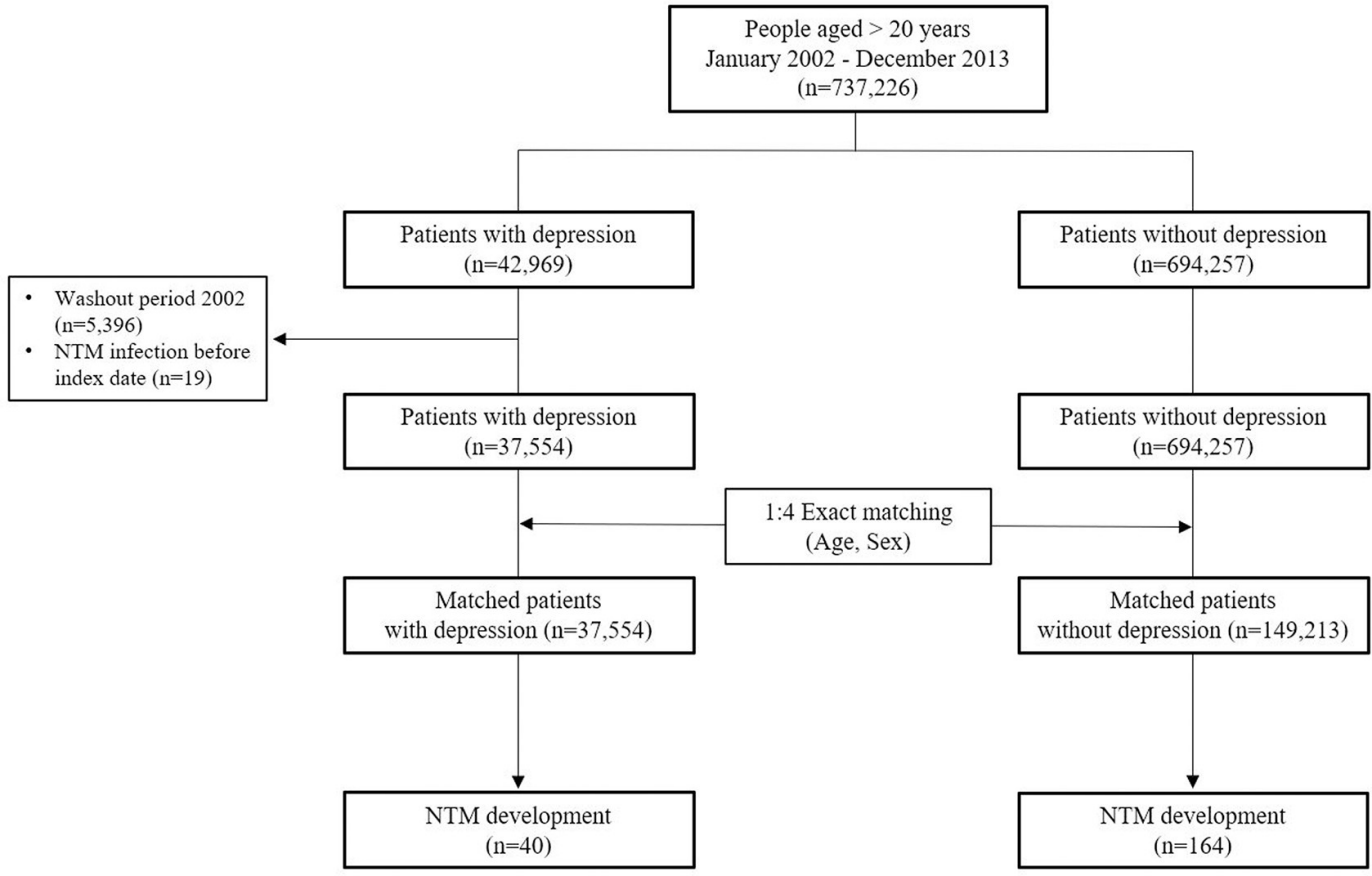
Flow chart of study population from the Korean National Health Insurance Service—National Sample Cohort.

Depression was defined using ICD-10 codes (F32.0, F32.1, F32.2, F32.3, F32.8, F32.9, F33.0, F33.1, F33.2, F33.8, and F33.9), and included patients who visited the clinic at least two times. The index date was defined as the first claim date for depression. To establish a matched cohort, the depression and non-depression groups were matched in terms of age and sex in the index date year in a ratio of approximately 1:4 using exact matching. To minimize selection bias, we used a random number order to select control participants for patients in the depression group. Finally, 37,554 patients in the depression group and 149,213 matched controls were included and followed-up until the development of NTM infection, death, or the end of the study period (Fig 1).

### Outcomes and covariates

The primary outcome was the incidence and risk of NTM infection in patients in the depression cohort versus those in the matched non-depression cohort. NTM infections were identified using ICD-10 codes (A31) and defined as patients with two or more claims diagnosed with the A31 code [3].

Data on sociodemographic characteristics, comorbidities, and other clinical characteristics were obtained from the NHIS database. Participants were divided into 14 age groups at 5-year intervals. Income groups were initially divided into 11 classes (class 0, lowest income; class 10, highest income) and reclassified into three classes (low, class 0–2; medium, class 3–7; high, class 8–10). The region of residence was divided into 16 areas according to administrative districts and categorized as urban (Seoul, Busan, Daegu, Incheon, Gwangju, Daejeon, and Ulsan) and rural (Gyeonggi, Gangwon, Chungcheongbuk, Chungcheongnam, Jeollabuk, Jeollanam, Gyeongsangbuk, Gyeongsangnam, and Jeju) regions. Pulmonary comorbidities (asthma, chronic obstructive pulmonary disease, bronchiectasis, and tuberculosis) and extrapulmonary comorbidities (diabetes and malignancies) were investigated using ICD-10 codes and defined as having two or more claims for disease-specific codes within 1 year preceding the index date.

### Statistical analysis

The general characteristics of the depression and non-depression groups were compared using the chi-square test for categorical variables and independent t-test for continuous variables [16]. The incidence rate was calculated as 100,000 person-years (PY) and was stratified by age and sex. To compare the difference in incidence between the two groups, we used the Poisson regression model and presented the incidence rate ratio and 95% confidence interval (95% CI). To account for the competing risk of death, the risk of development of NTM infection was estimated using the Fine and Gray regression model by calculating the subdistribution hazard ratios with 95% CI for the depression group compared to those of the non-depression group. The cumulative incidence rates between the two groups were compared using Gray’s test. Statistical analyses were performed using SAS version 9.4 (SAS Institute, Cary, NC, USA). Two-sided p < 0.05 was considered statistically significant.

### Ethical approval

This study was conducted in accordance with the 2008 Declaration of Helsinki and approved by the independent institutional review board (IRB) of the Severance Hospital (IRB number:4-2022-0761). No written informed consent was required as the patient records and information were anonymized and de-identified prior to the analyses. The requirement for informed consent was waived by the IRB of the Severance Hospital.

## Results

### Baseline characteristics

The baseline characteristics of the depression and non-depression groups before and after exact matching are presented in Table 1. The age- and sex-related characteristics did not significantly differ between the two groups after exact matching. The income and regional distribution of the study population was slightly different between the depression and non-depression groups after exact matching (p<0.001 and p = 0.025, respectively). Moreover, the number of patients with pulmonary and extrapulmonary comorbidities was significantly higher in the depression group than in the non-depression group (p<0.001 each). The mortality rate was also higher in the depression group than in the non-depression group (p<0.001).

**Table 1:** Baseline characteristics of the study population before and after exact matching.

Variables	Unmatched cohort			Matched cohort		
	Depression (n = 37,554)	Non-depression (n = 694,257)	p-value	Depression (n = 37,554)	Non-depression (n = 149,213)	p-value
**Sex**			< .0001			0.6371
Men	12,752 (33.96)	346,816 (49.95)		12,752 (33.96)	50,860 (34.09)	
Women	24,802 (66.04)	347,441 (50.05)		24,802 (66.04)	98,353 (65.91)	
**Age**			< .0001			0.9972
20–24	325 (0.87)	81,546 (11.75)		325 (0.87)	1,300 (0.87)	
25–29	1,450 (3.86)	82,362 (11.86)		1,450 (3.86)	5,800 (3.89)	
30–34	2,672 (7.12)	93,432 (13.46)		2,672 (7.12)	10,688 (7.16)	
35–39	3,252 (8.66)	84,684 (12.2)		3,252 (8.66)	13,003 (8.71)	
40–44	3,496 (9.31)	88,691 (12.77)		3,496 (9.31)	13,980 (9.37)	
45–49	3,986 (10.61)	67,997 (9.79)		3,986 (10.61)	15,941 (10.68)	
50–54	4,286 (11.41)	49,308 (7.1)		4,286 (11.41)	17,129 (11.48)	
55–59	3,648 (9.71)	39,081 (5.63)		3,648 (9.71)	14,568 (9.76)	
60–64	3,405 (9.07)	38,329 (5.52)		3,405 (9.07)	13,542 (9.08)	
65–69	3,583 (9.54)	28,293 (4.08)		3,583 (9.54)	14,198 (9.52)	
70–74	3,291 (8.76)	18,634 (2.68)		3,291 (8.76)	12,935 (8.67)	
75–79	2,285 (6.08)	11,716 (1.69)		2,285 (6.08)	8,904 (5.97)	
80–84	1,217 (3.24)	6,641 (0.96)		1,217 (3.24)	4,711 (3.16)	
≥85	658 (1.75)	3,543 (0.51)		658 (1.75)	2,514 (1.68)	
**Income**			< .0001			< .0001
Low	6,392 (17.02)	112,529 (16.21)		6,392 (17.02)	28,005 (18.77)	
Medium	14,867 (39.59)	325,328 (46.86)		14,867 (39.59)	62,044 (41.58)	
High	16,295 (43.39)	256,400 (36.93)		16,295 (43.39)	59,164 (39.65)	
**Region**			< .0001			0.025
Urban	17,002 (45.27)	334,821 (48.23)		17,002 (45.27)	68,516 (45.92)	
Rural	20,552 (54.73)	359,436 (51.77)		20,552 (54.73)	80,697 (54.08)	
**Pulmonary comorbidities**						
Asthma	-	-		1,861 (4.96)	5,242 (3.51)	< .0001
Chronic obstructive pulmonary disease	-	-		1,007 (2.68)	2,515 (1.69)	< .0001
Bronchiectasis	-	-		146 (0.39)	322 (0.22)	< .0001
Previous tuberculosis	-	-		154 (0.41)	434 (0.29)	< .0001
**Extrapulmonary comorbidities**						
Diabetes	-	-		3,943 (10.5)	12,405 (8.31)	< .0001
Malignancies	-	-		1,331 (3.54)	3,670 (2.46)	< .0001
**Mortality rate**	-	-		2,048 (5.96)	7,117 (5.55)	< .0001

### Association between NTM-PD and depression

**Fig 2:**
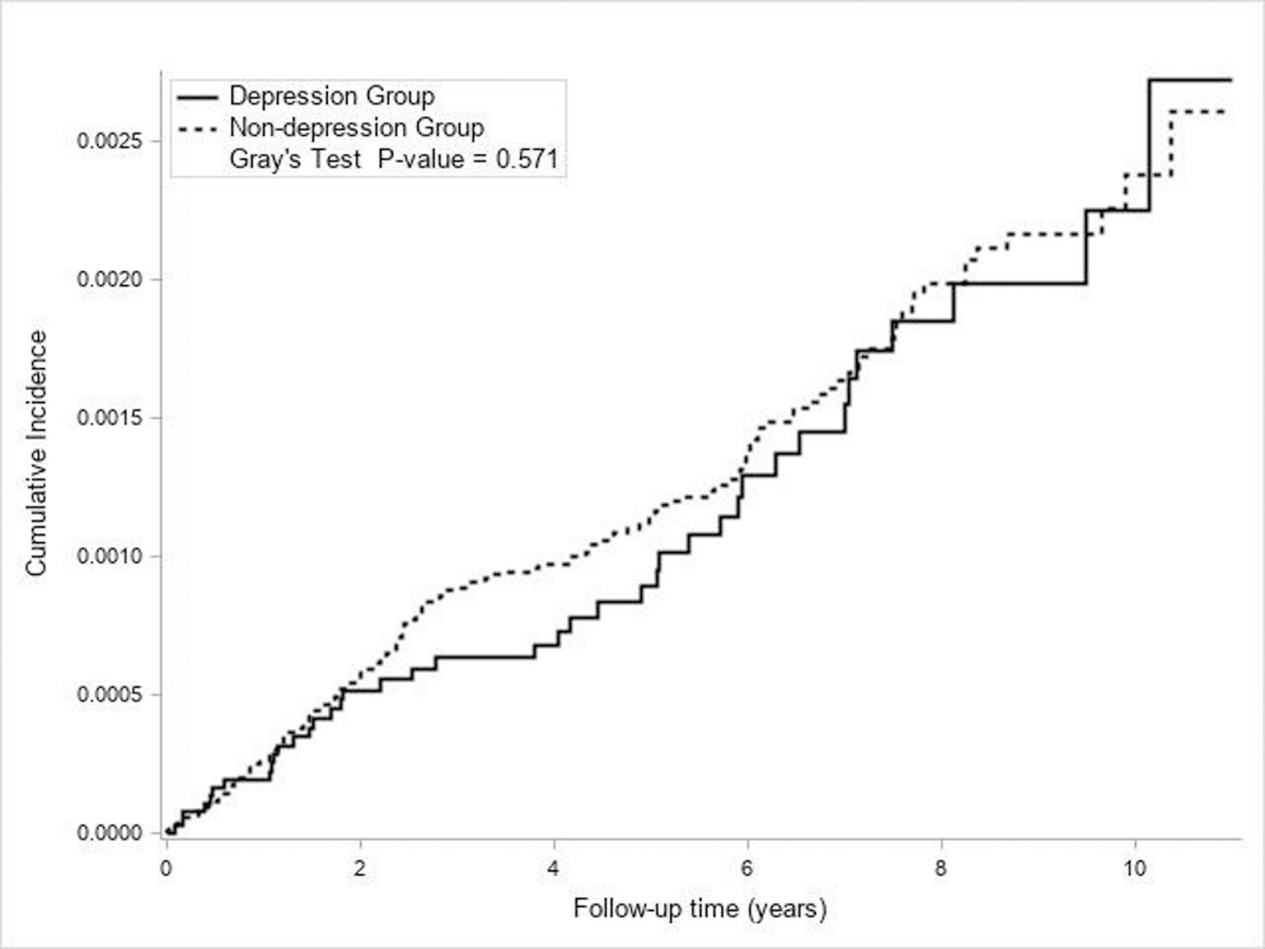
Cumulative incidence of NTM in the depression group and non-depression group for matched cohorts.

40 (22.2/100,000 PY) and 164 (24.5/100,000 PY) incident NTM infections occurred in the depression and non-depression groups, respectively. The cumulative incidence of NTM infection did not differ significantly between both groups during the follow-up period (p = 0.571, Fig 2).

The incidence rate of NTM infection was similar in men and women in both the depression (26.0 vs. 20.4 per 100,000 PY, p = 0.4594) and non-depression (29.2 vs. 22.3 per 100,000 PY, p = 0.0918) groups. The ≥70-year-old group had a higher incidence of NTM infection than that of the 20–49-year-old group in both the depression (56.3 vs. 6.1 per 100,000 PY, p<0.001) and non-depression groups (47.4 vs. 12.4 per 100,000 PY, p<0.001). However, the incidence of NTM infection did not significantly differ between the depression and non-depression groups according to sex and age (Table 2). After further adjusting for comorbidity, income, and region, the risk of NTM infection also did not significantly differ between the depression and non-depression groups (adjusted hazard ratio 0.83, 95% CI 0.58–1.17, Table 3).

**Table 2:** Risks of NTM-PD measured by age and sex for matched cohorts.

Variables	Depression (n = 37,554)		Non-depression (n = 149,213)		Compared to non-depression group	
	NTM-PD (n = 40)	Incidence rate (95% CI)	NTM-PD (n = 164)	Incidence rate (95% CI)	IRR (95% CI)	HR (95% CI)
**Age**						
20–49	5 (12.5)	6.1 (2.5–14.6)	38 (23.17)	12.4 (9.0–17.0)	0.49 (0.19–1.25)	0.48 (0.19–1.23)
50–69	20 (50)	28.1 (18.1–43.5)	80 (48.78)	30.1 (24.2–37.5)	0.93 (0.57–1.52)	0.93 (0.57–1.52)
≥70	15 (37.5)	56.3 (33.9–93.3)	46 (28.05)	47.4 (35.5–63.2)	1.19 (0.66–2.13)	1.21 (0.68–2.15)
**Sex**						
Men	15 (37.5)	26 (15.7–43.1)	62 (37.8)	29.2 (22.8–37.5)	0.89 (0.51–1.56)	0.9 (0.51–1.58)
Women	25 (62.5)	20.4 (13.8–30.2)	102 (62.2)	22.3 (18.4–27.1)	0.92 (0.59–1.42)	0.91 (0.59–1.41)

*Abbreviations: NTM-PD, Nontuberculous mycobacterial pulmonary disease; Incidence rate (per 100,000 person-years); IRR, Incidence rate ratio; HR, Hazard ratio; CI, Confidence interval

**Table 3:** aHR for NTM-PD in the depression group compared to the non-depression group.

Variables	Matched cohort	
	IRR (95% CI)	aHR (95% CI)
Adjusted for		
Age, Sex	0.91 (0.64–1.28)	0.91 (0.64–1.28)
Age, Sex, Comorbidity	0.83 (0.59–1.17)	0.84 (0.59–1.18)
Age, Sex, Comorbidity, Income, Region	0.82 (0.58–1.16)	0.83 (0.58–1.17)

*Abbreviations: NTM-PD, Nontuberculous mycobacterial pulmonary disease; IRR, Incidence rate ratio; aHR, Adjusted hazard ratio; CI, Confidence interval

## Discussion

In this study, there was no significant difference in the incidence of NTM infections between the depression and non-depression groups. The age- and sex-stratified effects of NTM infection incidence were not significantly higher in people with depression than in those without depression.

To the best of our knowledge, this is the first study to investigate the incidence and associated risk of NTM infections in patients with depression. As the number of patients with depression increases worldwide, the importance of mental health problems is also emphasized in the field of NTM-PD [17]. Because NTM-PD has a prolonged disease course and requires long-term antibiotic treatment, mental health problems must be considered for optimal management. Previous studies have reported that chronic respiratory diseases or mental health problems, such as depression and anxiety, are comorbid with NTM-PD [10, 18, 19]. Depression is more prevalent in women than in men and in older patients than in younger patients, which is consistent with the risk factors for NTM-PD [20–22]. However, in our study, the incidence of NTM infection did not increase in the depression group compared to that in the non-depression group.

Epidemiological studies have shown that the prevalence and incidence of NTM-PD increase with age in most countries, including Korea [3, 23]. Similar to previous studies, our results also showed a higher incidence of NTM-PD in the ≥70-year-old group than that in the 20–49-year-old group in both the depression and non-depression groups. However, the incidence of NTM infections did not significantly differ between men and women. Although NTM-PD is generally more common in women, sex differences in the incidence of NTM-PD vary between studies. Some studies have reported a higher prevalence and incidence of NTM-PD in women; however, others have reported no differences between men and women [21, 24–26]. Our study identified no difference in the incidence of NTM infection because it was calculated in a depression group and a matched control group, not in the overall population.

The association of chronic lung diseases such as chronic obstructive pulmonary disease [7], tuberculosis [9], and depression has been much investigated compared to NTM-PD. The prevalence of depression was higher in patients with chronic lung disease compared to the general population. Although there was a lack of data for NTM-PD, there was data showing the relatively high prevalence of depressive symptoms/depression in NTM-PD [10, 11, 27]. However, it is still unclear what happens first, the severe lung disease symptoms or depression.

In general, it is argued that severe respiratory symptoms and lack of physical activity lead to mental disorders or symptoms. However, depression has been shown to result in various immunological alterations including a reduction in the percent of lymphocyte and memory T cell response and there were reports of high incidence of TB and pneumococcal disease in the depression group [14, 28, 29]. Immune cells and cytokines are modulated in patients with depression, and can impair immune responses [30, 31]. These altered host immune systems may be susceptible to NTM infection, and individuals with impaired immune systems are at increased risk of NTM infection [1, 32]. In addition, depression and NTM-PD shared similar risk factors including female sex, old age, and under-weight [20, 33, 34]. Therefore, we speculated that NTM infection may be increased in patients with depression even though we could not find evidence for the higher risk of NTM-PD development in the depression group. However, further studies on the association between NTM-PD and depression are warranted. Because we used the claim data and defined the diagnosis of depression and NTM-PD by administrative diagnostic codes, under- or over-diagnosis for NTM infection or depression could be possible. Second, we used the sample cohort for the analysis and the number of NTM infections was as small as 40 in the depression group, which may have contributed to the nonsignificant association between depression and NTM infection. In addition, depression has various ranges of disease severity. The association between depression and NTM infection could be different by the severity of depression, however, we could not estimate those relationships because of the small number of NTM infections. It may also have contributed to the nonsignificant association of depression and NTM infection in our study.

This study had several limitations. First, the NHIS-NSC database does not provide detailed personal information regarding clinical and lifestyle factors. Variables that could affect the incidence of NTM infection, such as host susceptibility or environmental factors, were not analyzed. However, we obtained data on pre-existing diseases and information reflecting lifestyle factors, such as income and region, and analyzed them by adjusting for these variables. Second, the NTM infection sites were not classified. We analyzed the data using an ICD-10 code (A31), which includes NTM-PD and extrapulmonary NTM infections. Although NTM may cause infections at any site, such as the skin and joints, NTM-PD is the most common manifestation and was the basis for this analysis [35].

The incidence of NTM infections in patients with depression was not significantly higher than that in patients without depression. However, due to the small number of NTM infections, this study might have underestimated the differences between the two groups. Further studies are needed to address factors associated with NTM-PD in patients with depression.
